# Quantity of within-sport distance variety – what can pool swimmers and track runners learn from each other?

**DOI:** 10.3389/fspor.2024.1502758

**Published:** 2024-12-04

**Authors:** Dennis-Peter Born, Jenny Lorentzen, Glenn Björklund, Jesús J. Ruiz-Navarro

**Affiliations:** ^1^ Swiss Development Hub for Strength and Conditioning in Swimming, Swiss Swimming Federation, Worblaufen, Switzerland; ^2^ Department for Elite Sport, Swiss Federal Institute of Sport Magglingen, Magglingen, Switzerland; ^3^ Faculty of Science and Medicine, University of Fribourg, Fribourg, Switzerland; ^4^ Swedish Winter Sports Research Centre, Mid Sweden University, Östersund, Sweden; ^5^ Aquatics Lab, Department of Physical Education and Sports, Faculty of Sport Sciences, University of Granada, Granada, Spain

**Keywords:** adolescence, competition, diversification, elite athlete, long-term athlete development, sampling, talent

## Abstract

**Objective:**

To determine the relationship between success at peak performance age and *quantity of within-sport distance variety* and compare the *dose-time-effect* between swimming and track running by determining probability of becoming an international-class female athlete based on the number of different race distances the athletes compete in each year throughout their development process.

**Methods:**

Race times of female Tier 2 to Tier 5 freestyle pool swimmers (*n* = 2,778) and track runners (*n* = 9,945) were included in the present study. All athletes were ranked according to their personal best at peak performance age. Subsequently, number of different race distances during each year were retrospectively extracted from peak performance to early junior age. Personal best performance points at peak performance age were correlated with the number of different race distances across the various age categories. Poisson distribution determined the dose-time-effect of becoming an international-class athlete based on the number of different swimming strokes.

**Results:**

At peak performance age, correlation analysis showed a larger within-sport distance variety for higher ranked athletes, particularly for track runners (*r* ≤ 0.35, *P* < 0.001). Despite reaching statistical significance, the effects were small to moderate. While swimmers showed a generally larger within-sport distance variety than track runners, Poisson distribution revealed a dose-time-effect for the probability of becoming an international-class swimmer. Sprint and middle-distance swimmers benefit from competing in three race distances during junior age and a transition to two race distances at 17–18, 18–19, 20–21 and 25–26 years of age for 50 m, 100 m, 200 m and 400 m races, respectively. Long-distance swimmers should maintain three different race distances throughout peak performance age. Probability analysis showed a consistent benefit of competing in one or two race distances for 100 m, 200 m, 400 m and 800 m track runners.

**Conclusion:**

Within-sport distance variety is not a continuum but an ever-evolving process throughout the athletes' careers. While swimmers generally show larger variety than track runners, the progressive specialization towards peak performance age improves success chances to become an international-class swimmer.

## Introduction

Sport specialization is a heavily discussed topic among talent development experts: as such, some previous studies showed the benefits of sport variety during junior age for success at adult age (1–3). Other studies, however, found that specialization and performance level during junior age are related to adult success (4, 5). These conflicting findings may result from different methodological approaches evaluating involvement in different sports: *between-sport* variety (1–3), or involvement in different disciplines of a particular sport: *within-sport* variety (4, 5). Moreover, sport variety may not be a fixed variable, but rather an evolving factor throughout the development process of young talents. A previous study assessed within-sport variety regarding the involvement in different swimming strokes and showed a dose-time-effect for the probability of becoming an international-class swimmer. As such, a larger within-sport variety during early junior age and successive specialization in fewer swimming strokes were most beneficial for adult age success (6). However, the mentioned study only assessed involvement in different swimming strokes across the various 200 m events and warrants further investigations into the dose-time-effect of involvement in various race distances, i.e., sprint, middle- and long-distance events.

A recent study compared development of performances and race times of athletes' main and secondary events (*quality of variety*) in swimming and track running (7). These two sports have comparable conditions during competitions: (1) standardized distances on a flat course with limited environmental effects and electronical time measurements (8, 9), (2) time-wise equal race lengths (10, 11) and (3) similar physiological and metabolic demands (12–16). Despite these similarities, training regimes are substantially different between the two sports. Track runners emphasize under-distance and high-intensity training (17–19), while swimmers typically rely on long aerobic sets and over-distance training (20–22). Since *quantity of variety* has never been investigated in, nor compared between swimming and track running, determining the dose-time-effect of the number of different race distances the athletes compete in each year may provide deeper insights into the topic of variety and new inputs for prevailing training and development strategies.

Since female study participants are traditionally underrepresented in sport science articles (23), recent editorials and changes in journal policies demand more studies with particular attention to female athletes (24–26). This is particularly justified in swimming, since the considerably different anthropometrics and biological maturation of female compared to male athletes affect development of swimming performance (27, 28) and long-term athlete development programs (29). Due to a lower center of mass, slightly higher and better distributed body fat content even before puberty, and a more hydrodynamic body shape, female swimmers experience a lower leg sinking torque (30–33). Their greater joint mobility also contributes to swimming performance (34, 35). The more advantageous body composition and improved buoyancy allows female swimmers to focus on the development of key technical elements related to propulsion, such as hydrodynamic lift, catch of the water at the beginning of the arm stroke, and rotation along the longitudinal axis, at a younger age than males (36–39). Moreover, on average, growth velocity peaks two years earlier in females – specifically by the age of 11.9 ± 1.0 years compared to 14.1 ± 1.1 years in males (40).

As a result, female swimmers perform closer to the world record at a younger age, but also show an earlier performance plateau towards peak performance age (41, 42). Sex-specific analyses are warranted to assess all facets of the complexity of within-sport variety across the various race distances and improve long-term athlete development of female swimmers and track runners. Therefore, the aims of the present study were to (1) determine the relationship between success at peak performance age and *quantity of within-sport distance variety* and (2) compare the *dose-time-effect* between female swimmers and track runners by determining probability of becoming an international-class female athlete based on the number of different race distances the athletes compete in each year. The hypotheses were that higher ranked swimmers show a larger within-sport distance variety, however, with a dose-time-effect that allows for successive specialization towards peak performance age. Due to the over-distance oriented training, swimmers are expected to show a generally larger within-sport distance variety compared to track runners.

## Methods

### Subjects

Race times from officially licensed competitions with electronical time measurements were provided by the databases of the European swimming (43) and World athletics federations (44). A total of *n* = 2,778 individual female freestyle pool swimmers and *n* = 9,945 individual female track runners were included in the present study. Tier 2 to Tier 5 athletes (45) with >550 performance points at peak performance age (as described in detail later) were included. No explicit written informed consent was required, as only publicly available race times were included and analyzed anonymously. The study was preapproved by the institutional review board of the Swiss Federal Institute of Sport Magglingen (Reg.-Nr. 222_LSP_Born_03_2024) and conducted according to the guidelines of the World medical association for medical studies involving human subjects (Declaration of Helsinki).

### Procedure

All athletes, who were still competing at peak performance age, were ranked based on their personal best in the respective race distance. Subsequently, the number of different race distances during each year were retrospectively extracted from peak performance age throughout adolescence until early junior age (13 years of age). Ranking at peak performance age was considered as the dependent and number of different race distances the athletes competed in each year as the independent variable.

The ranking was based on performance points calculated as the division of the specific event's world record time by an individual swimmer's race time, then to the power of three, and multiplied by one thousand (9). As such, performance points can range from 1,000 (equal to the prevailing world record) to theoretically zero. However, since the present study aimed to find contributing factors to high-performance sports, only athletes reaching >550 performance points as their personal best at peak performance age (regional-class level) were included (46). The point system of the world governing body of swimming was used for both sports, since the study was conducted from the perspective of swimming with the aim of taking insights from land-based sports to further develop aquatic sports.

Only long-course (50 m pool length) freestyle swimming events (50 m, 100 m, 200 m, 400 m, 800 m, 1,500 m) and running events held on 400 m tracks (100 m, 200 m, 400 m, 800 m, 1,500 m, 3,000 m, 5,000 m, 10,000 m) were included. Freestyle was chosen, since this swimming stroke encompasses the widest range of race distances (6 vs. 3 in the other swimming strokes) (9). As such, the number of different race distances ranged from 1 to 6 in swimming and from 1 to 8 in running. To facilitate data interpretation and reduce multiple comparisons for the correlation analysis, the numbers of different race distances were averaged across two-year age categories, i.e., 13–14, 15–16, 17–18, 19–20, 21–22, 23–30 (23+) years of age. Peak performance age was set at 23–30 years of age based on previous research studies (47, 48) and the newly introduced U23 European junior championships, which should help swimmers transition from international junior to adult championships (49).

### Data analysis

To rank athletes at peak performance age, race times from the 2016–2023 databases were used. The retrospective tracking of the number of different race distances was conducted also using the 2006–2015 databases. Initial data extraction from the databases, calculation of performance points, establishment of ranking at peak performance age, and retrospective extraction of the number of different race distances per year were conducted in Python (version 3.11.5, Python Software Foundation, Beaverton, USA) using the “pandas” library for data analysis (version 2.2.1, pandas-dev/pandas, Zenodo, Genève, Switzerland). All subsequent data handling, including the calculation of probabilities using Poisson distribution, was conducted with Microsoft Excel 365 (version 2406, Microsoft Corporation, Redmond, WA, USA). The data analysis was coded by an experienced data scientist, holding a master's degree and PhD. The procedures were validated by another independent data analyst and the other scientists involved in the study.

### Statistical analysis

The *relationship* between ranking at peak performance age and within-sport distance variety, i.e., number of different race distances the athletes competed in each year, within the various age categories was assessed with Pearson's correlation coefficient (*r*). Spearman's *rho* was calculated if Q-Q plot or Shapiro-Wilk test showed non-normally distributed data. The magnitude of the correlation coefficients were interpreted as follows: trivial (<0.10), small (0.10–0.29), moderate (0.30–0.49), high (0.50–0.69), very high (0.70–0.89) and practically perfect (>0.90) (50, 51). Since the present study evaluates a population rather than a sample, i.e., all swimmers and runners still competing at peak performance age with >550 performance points, a *post-hoc* rather than *à priori* power analysis was conducted with G*Power (version 3.1.9.7). Using the point biserial model for two-tailed correlations, coefficients were used for effect sizes with their corresponding alpha-levels and sample sizes. Underpowered correlations with a statistical power <0.80 were disregarded from the data interpretation, regardless of their effect size or statistical significance (52).

The *dose-time-effect* of within-sport distance variety across the development process from junior to adult age was determined using Poisson distribution for international-class athletes [personal best of >750 performance points at peak performance age (46)]. The Poisson distribution reveals the probability that an independent event occurs, such as becoming an international-class athlete at peak performance age, based on the number of different race distances the athletes competed in each year. The likelihood of an independent event occurring is expressed between 0 and 1 for each point on a constant time scale using the probability mass function. Statistical analyses were conducted with JASP statistical software package (version 0.19, JASP-Team, University of Amsterdam, Amsterdam, The Netherlands). An alpha-level of 0.05 indicated statistical significance.

## Results

Table 1 shows the correlation analysis between personal best performance points at peak age (in the respective race distance) and number of different race distances the athletes competed in during the various age categories. Although correlations are highly significant (*P* < 0.001), magnitude of the coefficients are small to moderate. For swimmers, the effects increase towards peak performance age and show that higher ranking is associated with larger variety. Sprint and long-distance swimming events, i.e., 50 m and 1,500 m, showed fewer significant effects. In contrast, runners indicate highly significant effects over all race distances for the 19–20 year and older age categories.

**Table 1:** Correlation analysis between personal best performance points at peak age (in the respective race distance) and number of different race distances the athletes competed in during the various age categories. The correlation analysis included female world-class finalists, international-, national- and regional-class swimmers or runners (>550 performance points as personal best at peak performance age).

		Age categories [years]					
		13–14	15–16	17–18	19–20	21–22	23+
Swimmers							
50 m	*n* = 1,875	0.01	0.00	-0.01	0.04	0.05*	0.10***
100 m	*n* = 1,825	0.04	0.03	0.02	0.04	0.11***	0.10***
200 m	*n* = 1,333	0.02	0.06*	0.06	0.07*	0.14***	0.14***
400 m	*n* = 822	0.08	0.08*	0.10**	0.11**	0.20***	0.18***
800 m	*n* = 518	0.08	0.06	0.14**	0.15**	0.17***	0.10*
1,500 m	*n* = 275	0.05	0.03	0.09	0.00	0.04	–0.04
Runners							
100 m	*n* = 3,435		0.18***	0.22***	0.25***	0.26***	0.23***
200 m	*n* = 4,068		0.21***	0.28***	0.31***	0.35***	0.35***
400 m	*n* = 2,893		0.11**	0.21***	0.23***	0.26***	0.27***
800 m	*n* = 2,048		0.08	0.12***	0.22***	0.23***	0.18***
1,500 m	*n* = 2,581		0.14**	0.12***	0.17***	0.19***	0.29***
3,000 m	*n* = 1,274		0.22**	0.10*	0.19***	0.18***	0.28***
5,000 m	*n* = 2,033		0.03	0.09*	0.18***	0.14***	0.22***
10,000 m	*n* = 1,130				0.15***	0.11**	0.10***

Level of significance: *P < 0.05, **P < 0.01, ***P < 0.001.

Correlation coefficients with low statistical power (<0.80) are marked with gray color.

**Figure 1:**
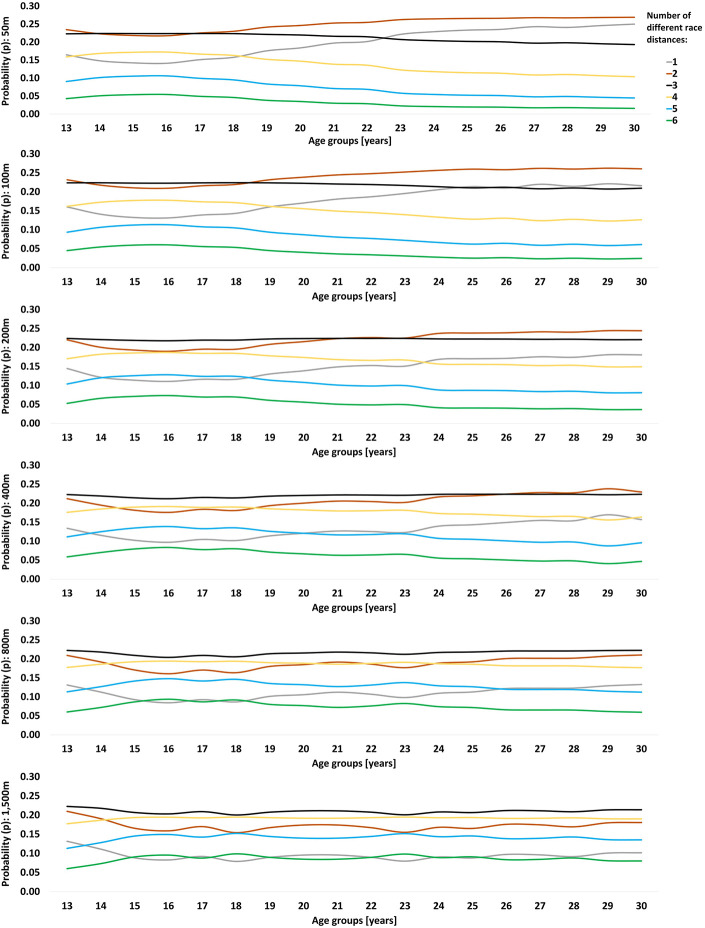
Dose-time-effect as probability (*p*) of becoming an international-class female swimmer based on the number of different race distances per year throughout adolescence until peak performance age. International-class was defined as a personal best of >750 performance points at peak performance age.

**Figure 2:**
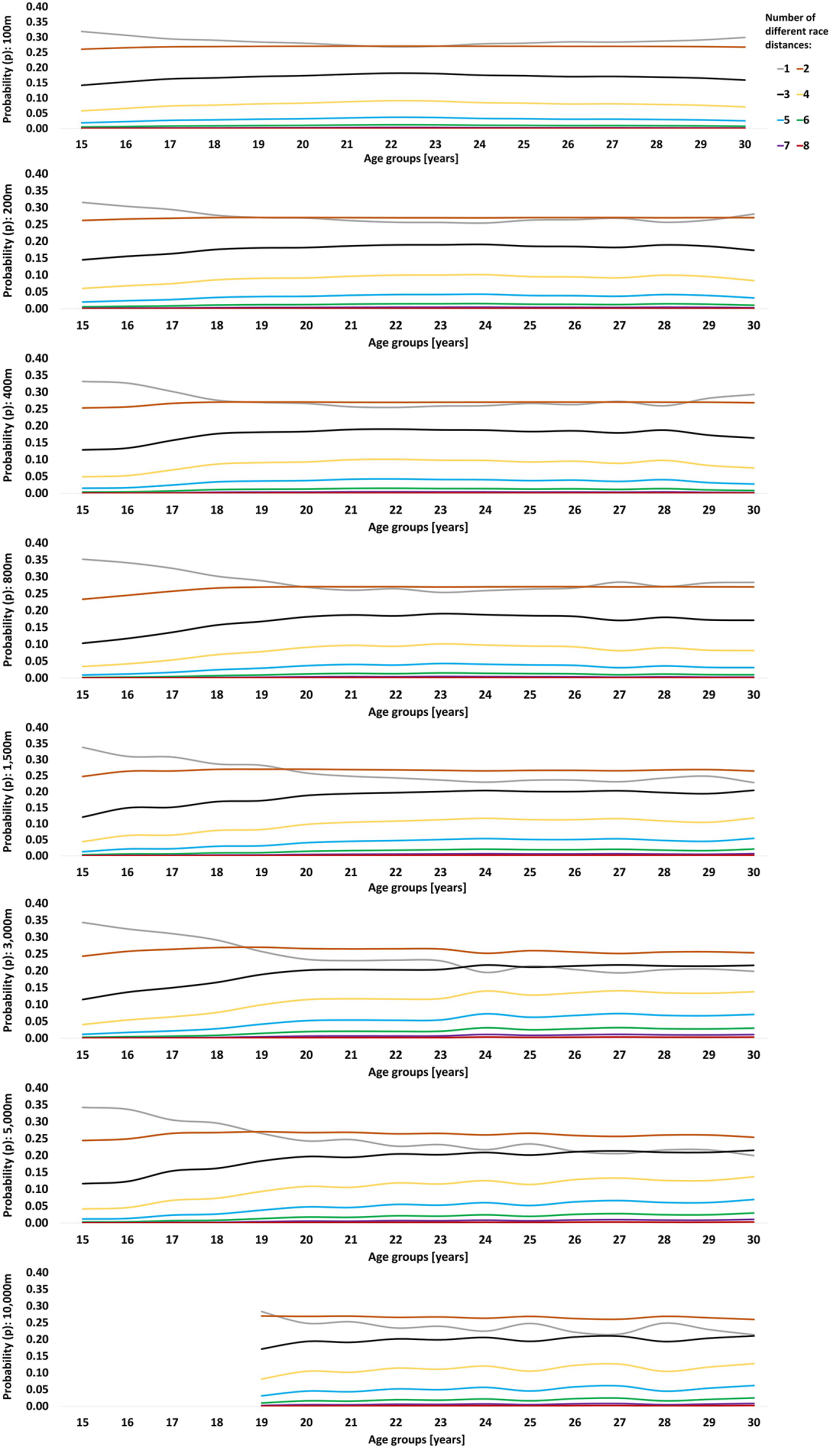
Dose-time-effect as probability (*p*) of becoming an international-class female track runner based on the number of different race distances per year throughout adolescence until peak performance age. International-class was defined as a personal best of >750 performance points at peak performance age.

The probabilities of becoming an international-class athlete at peak performance age are indicated in Figures 1, 2. Sprint and middle-distance swimmers benefit from competing in three race distances during junior age with a later transition to two race distances. This transition point occurs at an older age the longer the race distances, i.e., 17–18, 18–19, 20–21 and 25–26 years of age for 50 m, 100 m, 200 m and 400 m races, respectively, indicating that long-distance swimmers (800 m and 1,500 m) should maintain three different race distances throughout peak performance age. Low probabilities of becoming an international-class athlete were evident when competing in five to six race distances. Competing in only one race distance showed the second highest probability of becoming an international-class swimmer at 22–23 years of age for 50 m races. The advantage of competing in a single race distance diminishes as the swimming race distances increase. For track runners competing in 100 m, 200 m, 400 m or 800 m, probability analysis showed a consistent benefit of competing in one or two race distances. Long-distance track runners benefit from two race distances with three or a single race distance showing the second highest probabilities of becoming an international-class athlete.

Tables 2, 3 show the most common race distance combinations for all race distances and age categories. At peak performance age, 50 m, 100 m and 200 m swimmers typically compete in all those three race distances (50 m–200 m), the 400 m and 800 m swimmers compete in all available race distances (50 m–1,500 m), and the 1,500 m swimmers in all 200 m and longer races (200 m–1,500 m). Track runners typically compete in the neighboring race distances of their main event. There are few sprinters who also compete in middle-distance races, while 800 m runners commonly also compete in the 400 m distance. In detail, most 400 m runners (47.3%) additionally compete in 100 m and 200 m races but only a few (8.8%) compete in 800 m races, while the most common combination (28.9%) of the 800 m runners also involves 400 m races. Whereas 800 m runners compete in races of no longer than 1,500 m, the 1,500 m and long-distance runners commonly compete in both middle- and long-distance races.

**Table 2:** The three most common combinations of race distances for 50 m, 100 m, 200 m, 400 m, 800 m and 1,500 m female freestyle *swimmers* with >750 performance points at peak performance age across the various age categories.

	Age categories [years]					
	13–14	15–16	17–18	19–20	21–22	23+
50 m	**27.3%^[50, 100, 200]^**	**30.8%^[50, 100, 200]^**	**38.2%^[50, 100, 200]^**	**44.3%^[50, 100, 200]^**	**45.9%^[50, 100, 200]^**	**38.3%^[50, 100, 200]^**
	23.8%^[50, 100, 200, 400, 800]^	22.9%^[50, 100, 200, 400]^	25.2%^[50, 100, 200, 400]^	25.7%^[50, 100]^	30.8%^[50, 100]^	34.5%^[50, 100]^
	20.3%^[50, 100, 200, 400]^	20.4%^[50, 100]^	19.3%^[50, 100]^	16.1%^[50, 100, 200, 400]^	11.9%^[50, 100, 200, 400]^	16.2%^[50, 100, 200, 400]^
100 m	**26.1%^[50, 100, 200, 400, 800]^**	**25.0%^[50, 100, 200]^**	**32.7%^[50, 100, 200]^**	**37.3%^[50, 100, 200]^**	**44.3%^[50, 100, 200]^**	**36.8%^[50, 100, 200]^**
	21.6%^[50, 100, 200]^	23.3%^[50, 100, 200, 400]^	27.2%^[50, 100, 200, 400]^	19.6%^[50, 100, 200, 400]^	19.9%^[50, 100]^	24.2%^[50, 100]^
	19.1%^[50, 100, 200, 400]^	20.8%^[50, 100, 200, 400, 800]^	13.7%^[50, 100, 200, 400, 800]^	17.7%^[50, 100]^	15.4%^[50, 100, 200, 400]^	19.6%^[50, 100, 200, 400]^
200 m	**27.3%^[50, 100, 200, 400, 800]^**	**22.5%^[50, 100, 200, 400, 800]^**	**19.4%^[50, 100, 200, 400]^**	**21.2%^[50, 100, 200, 400]^**	**23.8%^[50, 100, 200]^**	**22.1%^[50, 100, 200]^**
	17.2%^[50, 100, 200]^	19.5%^[50, 100, 200, 400]^	18.0%^[50, 100, 200, 400, 800]^	19.8%^[50, 100, 200]^	16.8%^[50, 100, 200, 400]^	21.2%^[50, 100, 200, 400]^
	16.0%^[50, 100, 200, 400]^	18.5%^[50, 100, 200, 400, 800, 1500]^	17.5%^[50, 100, 200]^	13.4%^[50, 100, 200, 400, 800]^	12.4%^[50, 100, 200, 400, 800]^	11.6%^[50, 100, 200, 400, 800]^
400 m	**35.4%^[50, 100, 200, 400, 800]^**	**30.6%^[50, 100, 200, 400, 800, 1500]^**	**27.3%^[50, 100, 200, 400, 800, 1500]^**	**15.5%^[50, 100, 200, 400, 800, 1500]^**	**15.5%^[200, 400, 800, 1500]^**	**15.8%^[50, 100, 200, 400, 800, 1500]^**
	12.0%^[100, 200, 400, 800]^	21.3%^[50, 100, 200, 400, 800]^	17.5%^[50, 100, 200, 400, 800]^	14.5%^[50, 100, 200, 400, 800]^	14.5%^[50, 100, 200, 400, 800, 1500]^	15.4%^[50, 100, 200, 400]^
	11.2%^[50, 100, 200, 400, 800, 1500]^	8.74%^[50, 100, 200, 400]^	12.8%^[100, 200, 400, 800, 1500]^	13.5%^[100, 200, 400, 800, 1500]^	14.0%^[50, 100, 200, 400, 800]^	14.5%^[50, 100, 200, 400, 800]^
800 m	**37.9%^[50, 100, 200, 400, 800]^**	**33.3%^[50, 100, 200, 400, 800, 1500]^**	**32.3%^[50, 100, 200, 400, 800, 1500]^**	**21.8%^[200, 400, 800, 1500]^**	**25.3%^[200, 400, 800, 1500]^**	**22.6%^[50, 100, 200, 400, 800, 1500]^**
	13.7%^[100, 200, 400, 800]^	15.5%^[50, 100, 200, 400, 800]^	19.0%^[50, 100, 200, 400, 800]^	21.1%^[100, 200, 400, 800, 1500]^	16.9%^[50, 100, 200, 400, 800, 1500]^	20.2%^[200, 400, 800, 1500]^
	12.6%^[50, 100, 200, 400, 800, 1500]^	14.8%^[100, 200, 400, 800, 1500]^	16.9%^[100, 200, 400, 800, 1500]^	20.4%^[50, 100, 200, 400, 800, 1500]^	16.1%^[100, 200, 400, 800, 1500]^	14.7%^[50, 100, 200, 400, 800]^
1,500 m	**35.7%^[50, 100, 200, 400, 800, 1500]^**	**50.0%^[50, 100, 200, 400, 800, 1500]^**	**47.0%^[50, 100, 200, 400, 800, 1500]^**	**33.3%^[200, 400, 800, 1500]^**	**38.9%^[200, 400, 800, 1500]^**	**33.3%^[200, 400, 800, 1500]^**
	21.4%^[100, 200, 400, 800, 1500]^	26.9%^[100, 200, 400, 800, 1500]^	29.4%^[200, 400, 800, 1500]^	29.1%^[100, 200, 400, 800, 1500]^	25.9%^[100, 200, 400, 800, 1500]^	32.2%^[50, 100, 200, 400, 800, 1500]^
	21.4%^[200, 400, 800, 1500]^	17.3%^[200, 400, 800, 1500]^	22.0%^[100, 200, 400, 800, 1500]^	26.3%^[50, 100, 200, 400, 800, 1500]^	22.0%^[50, 100, 200, 400, 800, 1500]^	17.7%^[100, 200, 400, 800, 1500]^

Bold values indicate the most common combination of race distances for the particular distance and age category.

**Table 3:** The three most common combinations of race distances for 100 m, 200 m, 400 m, 800 m, 1,500 m, 3,000 m, 5,000 m and 10,000 m female *track runners* with > 750 performance points at peak performance age across the various age categories.

	Age categories [years]				
	15–16	17–18	19–20	21–22	23+
100 m	**66.0%^[100, 200]^**	**72.1%^[100, 200]^**	**68.7%^[100, 200]^**	**69.3%^[100, 200]^**	**60.5%^[100, 200]^**
	22.4%^[100]^	14.6%^[100, 200, 400]^	19.9%^[100, 200, 400]^	23.1%^[100, 200, 400]^	33.0%^[100, 200, 400]^
	11.4%^[100, 200, 400]^	13.2%^[100]^	10.9%^[100]^	7.3%^[100]^	6.1%^[100]^
200 m	**53.0%^[100, 200]^**	**56.0%^[100, 200]^**	**51.5%^[100, 200]^**	**50.8%^[100, 200]^**	**45.1%^[100, 200, 400]^**
	16.1%^[200, 400]^	21.9%^[100, 200, 400]^	28.1%^[100, 200, 400]^	30.5%^[100, 200, 400]^	41.3%^[100, 200]^
	15.1%^[100, 200, 400]^	14.2%^[200, 400]^	12.0%^[200, 400]^	13.9%^[200, 400]^	9.0%^[200, 400]^
400 m	**46.8%^[400]^**	**34.4%^[200, 400]^**	**38.1%^[200, 400]^**	**40.3%^[100, 200, 400]^**	**47.3%^[100, 200, 400]^**
	34.0%^[200, 400]^	30.5%^[100, 200, 400]^	33.6%^[100, 200, 400]^	37.2%^[200, 400]^	28.9%^[200, 400]^
	12.7%^[100, 200, 400]^	22.0%^[400]^	16.1%^[400]^	9.7%^[400]^	8.8%^[200, 400, 800]^
800 m	**45.9%^[800]^**	**29.6%^[800]^**	**27.9%^[800, 1500]^**	**32.1%^[800, 1500]^**	**28.9%^[400, 800, 1500]^**
	26.4%^[400, 800]^	29.2%^[400, 800]^	23.1%^[400, 800]^	22.5%^[400, 800, 1500]^	24.6%^[800, 1500]^
	17.2%^[800, 1500]^	15.6%^[800, 1500]^	20.9%^[400, 800, 1500]^	20.5%^[400, 800]^	11.6%^[400, 800]^
1,500 m	**31.9%^[1500]^**	**28.3%^[800, 1500]^**	**40.3%^[800, 1500]^**	**49.1%^[800, 1500]^**	**25.3%^[800, 1500, 3000, 5000]^**
	27.6%^[800, 1500]^	20.4%^[1500]^	16.5%^[400, 800, 1500]^	12.5%^[400, 800, 1500]^	24.6%^[800, 1500]^
	14.8%^[1500, 3000]^	18.1%^[400, 800, 1500]^	11.4%^[1500]^	8.7%^[800, 1500, 3000]^	10.3%^[800, 1500, 3000, 5000, 10,000]^
3,000 m	**38.8%^[3000]^**	**22.7%^[3000]^**	**33.3%^[1500, 3000, 5000]^**	**22.4%^[800, 1500, 3000, 5000]^**	**24.3%^[800, 1500, 3000, 5000]^**
	27.7%^[800, 1500, 3000]^	20.4%^[1500, 3000, 5000]^	15.0%^[800, 1500, 3000, 5000]^	22.4%^[1500, 3000, 5000]^	20.3%^[800, 1500, 3000, 5000, 10,000]^
	11.1%^[1500, 3000, 5000]^	18.1%^[800, 1500, 3000]^	13.3%^[1500, 3000, 5000, 10,000]^	13.7%^[3000, 5000, 10,000]^	19.5%^[1500, 3000, 5000, 10,000]^
5,000 m	**40.0%^[3000, 5000]^**	**38.8%^[1500, 3000, 5000]^**	**36.6%^[1500, 3000, 5000]^**	**18.0%^[1500, 3000, 5000]^**	**20.5%^[800, 1500, 3000, 5000, 10,000]^**
	30.0%^[1500, 3000, 5000]^	38.8%^[3000, 5000]^	15.5%^[1500, 3000, 5000, 10,000]^	15.0%^[800, 1500, 3000, 5000]^	20.0%^[800, 1500, 3000, 5000]^
	20.0%^[5000]^	11.1%^[5000]^	14.4%^[800, 1500, 3000, 5000]^	15.0%^[3000, 5000, 10,000]^	20.0%^[1500, 3000, 5000, 10,000]^
10,000 m			**37.0%^[1500, 3000, 5000, 10,000]^**	**48.2%^[5000, 10,000]^**	**27.2%^[1500, 3000, 5000, 10,000]^**
			29.6%^[3000, 5000, 10,000]^	26.7%^[3000, 5000, 10,000]^	23.9%^[3000, 5000, 10,000]^
			25.9%^[5000, 10,000]^	17.8%^[1500, 3000, 5000, 10,000]^	19.0%^[5000, 10,000]^

Bold values indicate the most common combination of race distances for the particular distance and age category.

## Discussion

The main findings of the present study are that the higher ranking of Tier 2 to Tier 5 female athletes correlates with a larger within-sport distance variety, especially towards peak performance age. As hypothesized, however, optimal variety for swimmers is not a continuum but an evolving process throughout the athletes' careers. As such, Poisson distribution shows the highest probability of becoming an international-class swimmer when competing in three to four race distances during junior age with a progressive reduction to two to three race distances towards peak performance age. International-class track runners generally show lower distance variety than swimmers. Competing in one to three race distances throughout the career appears most beneficial to become an international-class athlete. Within-sport distance variety is larger for long-distance athletes vs. sprinters for both runners and swimmers.

Correlation analysis revealed a small but significant relationship between a larger within-sport distance variety and success at peak performance age. However, increasing distance variety should not be interpreted as a causal effect that improves success chances, as better athletes may be capable of successfully competing in a larger number of race distances. For instance, at national championships and regional competitions, high-level athletes are important representatives for their home clubs. Due to their overall superior performance level, they typically compete in the relays, although these races do not necessarily cover their favorable or strongest distances. Moreover, the present study assessed quantity rather than quality of distance variety [refer to a previous article (7)] and therefore does not distinguish between primary and secondary race distances. Since high-level swimmers commonly use local competitions to compete in multiple secondary events for training purposes and to improve their competition routine (53), this may explain the larger within-sport distance variety in higher ranked athletes.

In both swimming and running, distance variety increases the longer the race distances. After crossing the transition point to mainly aerobic energy production in the 200 m swimming and 800 m running races, this metabolic energy system appears to be used in and adapted to longer race distances too (12, 13, 16, 54). The high reliance on over-distance training and aerobic sets in swimming particularly favors distance variety across middle- and long-distance races (20, 22). In contrast, the aerobic and anaerobic metabolic energy systems cannot be maximized at the same time and sprint swimmers may benefit from an earlier and larger degree of specialization to further improve performances, according to track sprinters' specialization pattern of 1–2 race distances. Additionally, sprint swimmers show specific stroke mechanics: the high cadence, extended elbow during the overwater movement, and fast hand entry at the beginning of the underwater phase of each arm stroke combined with the higher drag experience at such speeds require more energy and make the sprint-specific stroke mechanics uneconomically to maintain over longer race distances (55–60). To learn transfer this high cadence into propulsion, swimmers may have to accumulate a high amount of race pace specific training throughout their development process, hence sprinters transition from three to two race distances at a younger age than middle- and long distances swimmers, as revealed by the present study (Figure 1).

The present analysis showed that 50 m, 100 m and 200 m swimmers most commonly compete over all these three race distances at peak performance age (Table 2). The 50 m–200 m events provide the opportunity to compete in up to four different swimming strokes (9). As such, previous studies showed that freestyle swimmers commonly also compete in butterfly or backstroke events, allowing for a higher physiological specialization in the shorter events (6). In contrast, the most common combinations of race distances for 400 m and 800 m swimmers involve the full range of 50 m–1,500 m races. As 400 m and 800 m races provide little alternatives to freestyle, swimmers seem to spread their physiological capacity and increase their distance variety in order to maximize medal chances.

Although the present probability analysis revealed that sprinters showed the earliest transition from three to two race distances in swimming, track sprinters show an even earlier and larger degree of specialization on one to two race distances. The earlier performance plateau of female compared to male sprint swimmers (41) and the insights from track sprinters may motivate female sprint swimmers to focus even earlier on the specific development of their anaerobic energy system in order to maximize performance progression towards peak performance age. While female athletes are traditionally associated with lower trainability in muscular strength and power due to lower levels of testosterone and absolute muscle mass (61, 62), many other anabolic hormones and mechanical stress response induce substantial strength gains after resistance training (63–68). Besides the hypertrophic response, maximal strength gains due to neuro-muscular adaptations contribute significantly to swimming performance (69, 70). Therefore, taking resistance training seriously from an early stage of female swimmers' careers and developing specific resistance training and periodization protocols will help maximize progression of sprint performances towards peak performance age (71, 72).

### Limitations and future directions

The present study is limited to the quantification of variety based on the number of different race distances athletes competed in each year, without considering absolute performance differences between the various events. As discussed earlier, swimmers may compete in events outside their main race distance for training purposes, to represent their home club, or due to a lack of specific competition and development strategies. Therefore, the results of this study should be interpreted alongside previous research that accounts for performance differences between the main and secondary race distances, which is the quality of variety (7).

It is important to note that the correlation analysis, which associates larger variety with more success at peak performance age, does not indicate a causal effect. Since the correlation analysis involved Tier 2 to Tier 5 (45) swimmers (550–1,000 performance points at peak performance age), the results may be affected by low-level (regional-class) swimmers having not the same professional support and coaching staff like top-elite swimmers, hence a larger variety due to less specific development strategies. Therefore, the results of the probability analysis for international-class swimmers (>750 performance points) should be prioritized, which provides a more sophisticated analysis of the dose-time-effect of specialization throughout the swimmers' careers and showed the advantage of a large distance variety during early junior age but gradual specialization towards peak performance age.

Since the present analysis is limited to retrospective data, it is important to distinguish between descriptive data on the most common race distances in Tables 2, 3, and the probability analyses in Figures 1, 2. As best practice does not always provide the optimal development pathway for upcoming talents, future strategies should be developed based on a close interaction between practical experience and evidence-based knowledge (73). Therefore, specific training intervention studies are warranted to determine the causal effect of increased variety during junior age on success at peak performance age.

## Conclusion

The findings of the present study show that within-sport distance variety is not a continuum but an ever-evolving process throughout the athletes' careers. While swimmers generally show larger variety than track runners, the progressive specialization towards peak performance age improves success chances to become an international-class swimmer. Coaches and swimmers should establish their long-term development strategies based on the transition points at which the number of different race distances should be reduced together with the most commonly combined race distances. While long-distance athletes maintain a larger within-sport distance variety than sprinters, the insights from track running may motivate sprint swimmers to adopt an even earlier and higher degree of specialization for the optimal development of their anaerobic energy system and consequent implementation of resistance training from an early stage of their careers. The present study shows how the comparison between two sports with similar competition formats, but different training regimes, opens new perspectives and fuels the discussion about optimal long-term athlete development.

## Data Availability

Publicly available datasets were analyzed in this study. This data can be found here: Raw data are publicly available at the databases of the European swimming (43) and World athletics federations (44).

## References

[1] Güllich A. Many roads lead to Rome–developmental paths to Olympic gold in men’s field hockey. Eur J Sport Sci. (2014) 14:763–71. 10.1080/17461391.2014.905983 24707887

[2] Güllich A Macnamara BN Hambrick DZ. What makes a champion? Early multidisciplinary practice, not early specialization, predicts world-class performance. Perspect Psychol Sci. (2022) 17:6–29. 10.1177/1745691620974772 34260336

[3] Moesch K Elbe AM Hauge ML Wikman JM. Late specialization: the key to success in centimeters, grams, or seconds (cgs) sports. Scand J Med Sci Sports. (2011) 21:e282–90. 10.1111/j.1600-0838.2010.01280.x 21401722

[4] Post AK Koning RH Visscher C Elferink-Gemser MT. Multigenerational performance development of male and female top-elite swimmers-a global study of the 100 m freestyle event. Scand J Med Sci Sports. (2020) 30:564–71. 10.1111/sms.13599 31725946 PMC7028091

[5] Yustres I Del Cerro JS Martin R Gonzalez-Mohino F Logan O & Gonzalez-Rave JM. Influence of early specialization in world-ranked swimmers and general patterns to success. PLoS One. (2019) 14:e0218601. 10.1371/journal.pone.0218601 31220159 PMC6586317

[6] Born DP Lorentzen J Bjorklund G Stoggl T Romann M. Variation vs. specialization: the dose-time-effect of technical and physiological variety in the development of elite swimmers. BMC Res Notes. (2024) 17:48. 10.1186/s13104-024-06706-x 38355679 PMC10865614

[7] Born DP Romann M Lorentzen J Zumbach D Feldmann A Ruiz-Navarro JJ. Sprinting to the top: comparing quality of distance variety and specialization between swimmers and runners. Front Sports Act Living. (2024) 6:1431594. 10.3389/fspor.2024.1431594 39161627 PMC11330820

[8] World Athletics. Rules. Monaco: World Athletics (2024). Available online at: https://worldathletics.org/about-iaaf/documents/book-of-rules (accessed March 05, 2024).

[9] World Aquatics. Swimming Rules and Swimming Points. Lausanne: World Aquatics (2024). https://www.worldaquatics.com/rules/competition-regulations and https://www.worldaquatics.com/swimming/points (accessed March 10, 2024).

[10] World Aquatics. World records. Lausanne: World Aquatics (2024). Available online at: https://www.worldaquatics.com/swimming/records (accessed April 08, 2024).

[11] World Athletics. World records. Monaco: World Athletics (2024). Available online at: https://worldathletics.org/records/by-category/world-records (accessed April 08, 2024).

[12] Spencer MR Gastin PB. Energy system contribution during 200- to 1500-m running in highly trained athletes. Med Sci Sports Exerc. (2001) 33:157–62. 10.1097/00005768-200101000-00024 11194103

[13] Duffield R Dawson B Goodman C. Energy system contribution to 400-metre and 800-metre track running. J Sports Sci. (2005) 23:299–307. 10.1080/02640410410001730043 15966348

[14] Duffield R Dawson B Goodman C. Energy system contribution to 100-m and 200-m track running events. J Sci Med Sport. (2004) 7:302–13. 10.1016/S1440-2440(04)80025-2 15518295

[15] Massini DA Almeida TAF Macedo AG Espada MC Reis JF Alves FJB Sex-specific accumulated oxygen deficit during short- and middle-distance swimming performance in competitive youth athletes. Sports Med Open. (2023) 9:49. 10.1186/s40798-023-00594-4 37357246 PMC10290977

[16] Ribeiro J Figueiredo P Sousa A Monteiro J Pelarigo J Vilas-Boas JP VO(2) kinetics and metabolic contributions during full and upper body extreme swimming intensity. Eur J Appl Physiol. (2015) 115:1117–24. 10.1007/s00421-014-3093-5 25547736

[17] Haugen T Sandbakk O Seiler S Tonnessen E. The training characteristics of world-class distance runners: an integration of scientific literature and results-proven practice. Sports Med Open. (2022) 8:46. 10.1186/s40798-022-00438-7 35362850 PMC8975965

[18] Haugen T Sandbakk O Enoksen E Seiler S Tonnessen E. Crossing the golden training divide: the science and practice of training world-class 800- and 1500-m runners. Sports Med. (2021) 51:1835–54. 10.1007/s40279-021-01481-2 34021488 PMC8363530

[19] Haugen T Seiler S Sandbakk O Tonnessen E. The training and development of elite sprint performance: an integration of scientific and best practice literature. Sports Med Open. (2019) 5:44. 10.1186/s40798-019-0221-0 31754845 PMC6872694

[20] Pollock S Gaoua N Johnston MJ Cooke K Girard O Mileva KN. Training regimes and recovery monitoring practices of elite British swimmers. J Sports Sci Med. (2019) 18:577–85. 31427881 PMC6683628

[21] Nugent F Comyns T Kearney P Warrington G. Ultra-short race-pace training (USRPT) in swimming: current perspectives. Open Access J Sports Med. (2019) 10:133–44. 10.2147/OAJSM.S180598 31632163 PMC6789176

[22] Nugent FJ Comyns TM Warrington GD. Quality versus quantity debate in swimming: perceptions and training practices of expert swimming coaches. J Hum Kinet. (2017) 57:147–58. 10.1515/hukin-2017-0056 28713467 PMC5504587

[23] Costello JT Bieuzen F Bleakley CM. Where are all the female participants in sports and exercise medicine research? Eur J Sport Sci. (2014) 14:847–51. 10.1080/17461391.2014.911354 24766579

[24] Noordhof DA Janse De Jonge XAK Hackney AC De Koning JJ Sandbakk O. Sport-science research on female athletes: dealing with the paradox of concurrent increases in quantity and quality. Int J Sports Physiol Perform. (2022) 17:993–4. 10.1123/ijspp.2022-0185 35680118

[25] Mujika I Taipale RS. Sport science on women, women in sport science. Int J Sports Physiol Perform. (2019) 14:1013–4. 10.1123/ijspp.2019-0514 31484158

[26] Meyer T Cobley S. Addressing female underrepresentation in sport & exercise-related research: JSAMS policy for submitted studies (& subsequent considerations). J Sci Med Sport. (2024) 27:435–6. 10.1016/j.jsams.2024.05.018 38879218

[27] Miller AE Macdougall JD Tarnopolsky MA Sale DG. Gender differences in strength and muscle fiber characteristics. Eur J Appl Physiol Occup Physiol. (1993) 66:254–62. 10.1007/BF00235103 8477683

[28] Janssen I Heymsfield SB Wang ZM Ross R. Skeletal muscle mass and distribution in 468 men and women aged 18–88 yr. J Appl Physiol. (2000) 89:81–8. 10.1152/jappl.2000.89.1.81 10904038

[29] Swimming Canada. Long term athlete development strategy - swimming to win; winning for life! Ottawa: Swimming Canada (National swimming federation) (2008). Available online at: https://swimbc.ca/wp-content/uploads/SNC-LTAD-strategy-1.pdf (accessed November 08, 2024).

[30] Sandbakk O Solli GS Holmberg HC. Sex differences in world-record performance: the influence of sport discipline and competition duration. Int J Sports Physiol Perform. (2018) 13:2–8. 10.1123/ijspp.2017-0196 28488921

[31] Deurenberg P Pieters JJ Hautvast JG. The assessment of the body fat percentage by skinfold thickness measurements in childhood and young adolescence. Br J Nutr. (1990) 63:293–303. 10.1079/BJN19900116 2334665

[32] Caspersen C Berthelsen PA Eik M Pakozdi C Kjendlie PL. Added mass in human swimmers: age and gender differences. J Biomech. (2010) 43:2369–73. 10.1016/j.jbiomech.2010.04.022 20546755

[33] Zamparo P Cortesi M Gatta G. The energy cost of swimming and its determinants. Eur J Appl Physiol. (2020) 120:41–66. 10.1007/s00421-019-04270-y 31807901

[34] Tomkinson GR Carver KD Atkinson F Daniell ND Lewis LK Fitzgerald JS European normative values for physical fitness in children and adolescents aged 9–17 years: results from 2 779 165 eurofit performances representing 30 countries. Br J Sports Med. (2018) 52:1445–14563. 10.1136/bjsports-2017-098253 29191931

[35] Jagomagi G Jurimae T. The influence of anthropometrical and flexibility parameters on the results of breaststroke swimming. Anthropol Anz. (2005) 63:213–9. 10.1127/anthranz/63/2005/213 15962572

[36] Zhan J. M. Li T. Z. Chen X. B. Li Y. S. 2017. Hydrodynamic analysis of human swimming based on VOF method. Comput Methods Biomech Biomed Engin, 20, 645–52. 10.1080/10255842.2017.1284822 28127994

[37] Naemi R Easson WJ Sanders RH. Hydrodynamic glide efficiency in swimming. J Sci Med Sport. (2010) 13:444–51. 10.1016/j.jsams.2009.04.009 19540161

[38] Cortesi M Gatta G Michielon G Di Michele R Bartolomei S Scurati R. Passive drag in young swimmers: effects of body composition, morphology and gliding position. Int J Environ Res Public Health. (2020) 17(6):2002. 10.3390/ijerph17062002 32197399 PMC7142561

[39] Andersen JT Sinclair PJ Mccabe CB Sanders RH. Kinematic differences in shoulder roll and hip roll at different front crawl speeds in national level swimmers. J Strength Cond Res. (2020) 34:20–5. 10.1519/JSC.0000000000003281 31567840

[40] Koziel SM Malina RM. Modified maturity offset prediction equations: validation in independent longitudinal samples of boys and girls. Sports Med. (2018) 48:221–36. 10.1007/s40279-017-0750-y 28608181 PMC5752743

[41] Born DP Lomax I Rüeger E Romann M. Normative data and percentile curves for long-term athlete development in swimming. J Sci Med Sport. (2022) 25:266–71. 10.1016/j.jsams.2021.10.002 34764012

[42] Born DP Stoggl T Lorentzen J Romann M Bjorklund G. Predicting future stars: probability and performance corridors for elite swimmers. J Sci Med Sport. (2024c) 27:113–8. 10.1016/j.jsams.2023.10.017 37968181

[43] Swimrankings.Net. (2024). Available online at: https://www.swimrankings.net/ (accessed February 15, 2024).

[44] World Athletics. Stats Zone. Monaco: World Athletics (2024). Available online at: https://worldathletics.org/stats-zone (accessed Jun 02, 2024).

[45] Mckay AKA Stellingwerff T Smith ES Martin DT Mujika I Goosey-Tolfrey VL Defining training and performance caliber: a participant classification framework. Int J Sports Physiol Perform. (2022) 17:317–31. 10.1123/ijspp.2021-0451 34965513

[46] Ruiz-Navarro JJ Lopez-Belmonte O Gay A Cuenca-Fernandez F Arellano R. A new model of performance classification to standardize the research results in swimming. Eur J Sport Sci. (2022) 23(4):478–88. 10.1080/17461391.2022.2046174 35193458

[47] Allen SV Vandenbogaerde TJ Hopkins WG. Career performance trajectories of Olympic swimmers: benchmarks for talent development. Eur J Sport Sci. (2014) 14:643–51. 10.1080/17461391.2014.893020 24597644

[48] Haugen TA Solberg PA Foster C Moran-Navarro R Breitschadel F Hopkins WG. Peak age and performance progression in world-class track-and-field athletes. Int J Sports Physiol Perform. (2018) 13:1122–9. 10.1123/ijspp.2017-0682 29543080

[49] European Aquatics. European U23 Swimming Championships. Nyon, Switzerland: European Aquatics (2023). Available online at: https://www.len.eu/get-to-know-the-european-u23-swimming-championships/ (accessed March 07, 2024).

[50] Hopkins W. A Scale Magnitude for Effect Statistics. Sportscience.org web page (2002). http://www.sportsci.org/resource/stats/effectmag.html (accessed June 03, 2024).

[51] Hopkins WG Marshall SW Batterham AM Hanin J. Progressive statistics for studies in sports medicine and exercise science. Med Sci Sports Exerc. (2009) 41:3–13. 10.1249/MSS.0b013e31818cb278 19092709

[52] Cohen J. Statistical Power Analysis for the Behavioral Sciences. Hillsdale, N.J.: L. Erlbaum Associates (1988).

[53] Born DP Lomax I Romann M. Variation in competition performance, number of races, and age: long-term athlete development in elite female swimmers. PLoS One. (2020) 15:e0242442. 10.1371/journal.pone.0242442 33206722 PMC7673509

[54] Gastin PB. Energy system interaction and relative contribution during maximal exercise. Sports Med. (2001) 31:725–41. 10.2165/00007256-200131100-00003 11547894

[55] Toussaint HM De Groot G Savelberg HH Vervoorn K Hollander AP Van Ingen Schenau GJ. Active drag related to velocity in male and female swimmers. J Biomech. (1988) 21:435–8. 10.1016/0021-9290(88)90149-2 3417695

[56] Narita K Nakashima M Takagi H. Developing a methodology for estimating the drag in front-crawl swimming at various velocities. J Biomech. (2017) 54:123–8. 10.1016/j.jbiomech.2017.01.037 28249682

[57] Born DP Schönfelder M Logan O Olstad BH Romann M. Performance development of European swimmers across the Olympic cycle. Front Sports Act Living. (2022) 4:894066. 10.3389/fspor.2022.894066 35755613 PMC9231649

[58] Barbosa TM Fernandes RJ Keskinen KL Vilas-Boas JP. The influence of stroke mechanics into energy cost of elite swimmers. Eur J Appl Physiol. (2008) 103:139–49. 10.1007/s00421-008-0676-z 18214521

[59] Valkoumas I Gourgoulis V. Sprint resisted swimming training effect on the swimmer’s hand orientation angles. J Biomech. (2024) 164:111991. 10.1016/j.jbiomech.2024.111991 38359622

[60] Samson M Monnet T Bernard A Lacouture P David L. Kinematic hand parameters in front crawl at different paces of swimming. J Biomech. (2015) 48:3743–50. 10.1016/j.jbiomech.2015.07.034 26433921

[61] Vingren JL Kraemer WJ Ratamess NA Anderson JM Volek JS Maresh CM. Testosterone physiology in resistance exercise and training: the up-stream regulatory elements. Sports Med. (2010) 40:1037–53. 10.2165/11536910-000000000-00000 21058750

[62] Cureton KJ Collins MA Hill DW Mcelhannon FM Jr. Muscle hypertrophy in men and women. Med Sci Sports Exerc. (1988) 20:338–44. 10.1249/00005768-198808000-00003 3173042

[63] Askow AT Merrigan JJ Neddo JM Oliver JM Stone JD Jagim AR Effect of strength on velocity and power during back squat exercise in resistance-trained men and women. J Strength Cond Res. (2019) 33:1–7. 10.1519/JSC.0000000000002968 30431534

[64] Crewther B Keogh J Cronin J Cook C. Possible stimuli for strength and power adaptation: acute hormonal responses. Sports Med. (2006) 36:215–38. 10.2165/00007256-200636030-00004 16526834

[65] Crewther B Cronin J Keogh J. Possible stimuli for strength and power adaptation: acute mechanical responses. Sports Med. (2005) 35:967–89. 10.2165/00007256-200535110-00004 16271010

[66] Staron RS Karapondo DL Kraemer WJ Fry AC Gordon SE Falkel JE Skeletal muscle adaptations during early phase of heavy-resistance training in men and women. J Appl Physiol. (1994) 76:1247–55. 10.1152/jappl.1994.76.3.1247 8005869

[67] Staron RS Leonardi MJ Karapondo DL Malicky ES Falkel JE Hagerman FC Strength and skeletal muscle adaptations in heavy-resistance-trained women after detraining and retraining. J Appl Physiol. (1991) 70:631–40. 10.1152/jappl.1991.70.2.631 1827108

[68] Kittilsen HT Goleva-Fjellet S Freberg BI Nicolaisen I Stoa EM Bratland-Sanda S Responses to maximal strength training in different age and gender groups. Front Physiol. (2021) 12:636972. 10.3389/fphys.2021.636972 33679448 PMC7925619

[69] Cormie P Mcguigan MR Newton RU. Developing maximal neuromuscular power: part 1–biological basis of maximal power production. Sports Med. (2011) 41:17–38. 10.2165/11537690-000000000-00000 21142282

[70] Crowley E Harrison AJ Lyons M. The impact of resistance training on swimming performance: a systematic review. Sports Med. (2017) 47:2285–307. 10.1007/s40279-017-0730-2 28497283

[71] Kissow J Jacobsen KJ Gunnarsson TP Jessen S Hostrup M. Effects of follicular and luteal phase-based menstrual cycle resistance training on muscle strength and mass. Sports Med. (2022) 52:2813–9. 10.1007/s40279-022-01679-y 35471634

[72] Rissanen J Walker S Pareja-Blanco F Hakkinen K. Velocity-based resistance training: do women need greater velocity loss to maximize adaptations? Eur J Appl Physiol. (2022) 122:1269–80. 10.1007/s00421-022-04925-3 35258681 PMC9012837

[73] Haugen T. 2021. Best-practice coaches: an untapped resource in sport-science research. Int J Sports Physiol Perform, 16, 1215–6. 10.1123/ijspp.2021-0277 34271549

